# COVID-19 and RAS: Unravelling an Unclear Relationship

**DOI:** 10.3390/ijms21083003

**Published:** 2020-04-24

**Authors:** Damiano D’Ardes, Andrea Boccatonda, Ilaria Rossi, Maria Teresa Guagnano, Francesca Santilli, Francesco Cipollone, Marco Bucci

**Affiliations:** Clinica Medica Institute, European Center of Excellence on Atherosclerosis, Hypertension and Dyslipidemia, “G. D’Annunzio” University of Chieti-Pescara, 66100 Chieti, Italyandrea.boccatonda@gmail.com (A.B.); rossilaria91@gmail.com (I.R.); guagnano@unich.it (M.T.G.); francesco.cipollone@unich.it (F.C.); mbucci@unich.it (M.B.)

**Keywords:** RAS, ACE, AT1R, ACE2, COVID-19, SARS-CoV-2

## Abstract

The renin-angiotensin system (RAS) plays a main role in regulating blood pressure and electrolyte and liquid balance. Previous evidence suggests that RAS may represent an important target for the treatment of lung pathologies, especially for acute respiratory distress syndrome and chronic fibrotic disease. The scientific community has recently focused its attention on angiotensin-converting enzyme (ACE) inhibitors and angiotensin receptor 1 (AT1R) inhibitors and their possible benefit/harms for patients infected by Coronavirus disease (COVID-19) who experience pneumonia, but there are still some doubts about the effects of these drugs in this setting.

## 1. The Physiological Basis of RAS 

The renin-angiotensin system (RAS) is known to play an important role in regulating blood pressure and electrolyte and water balance.

**Figure 1:**
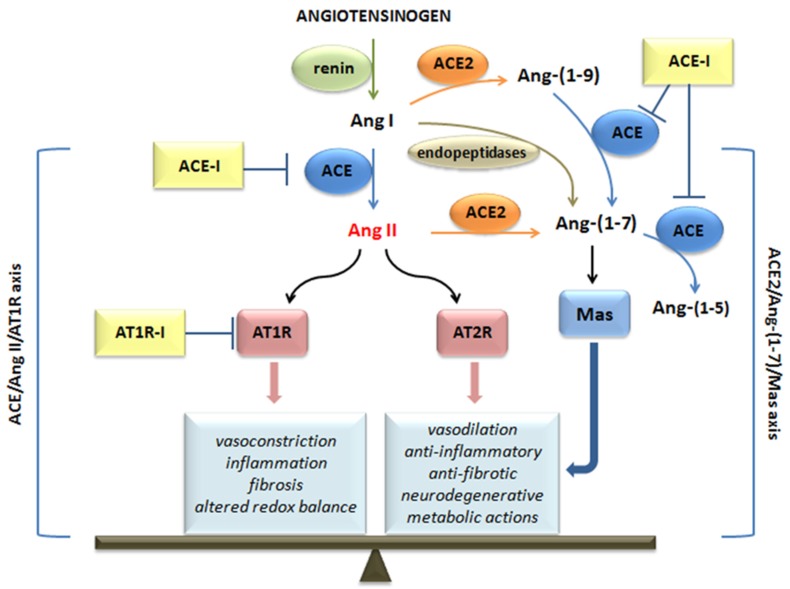
The renin-angiotensin system (RAS) cascade and angiotensin-converting enzyme (ACE) inhibitors and angiotensin receptor 1 (AT1R) inhibitors action. Ang I: angiotensin I; Ang II: angiotensin II; ACE: angiotensin-converting enzyme; ACE2: angiotensin-converting enzyme 2; ATR1: angiotensin II receptor type 1; ATR2: angiotensin II receptor type 2; ACE-I: ACE inhibitors; AT1R-I: angiotensin receptor 1 inhibitors. → transformation; ┤ inhibition; 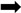 effects mediated.

The RAS pathway consists of a cascade of proteases producing some bioactive molecules [1]. Angiotensinogen is mainly released by the liver and then cleaved by renin, which is secreted by the juxtaglomerular cells in the kidney, thus generating the decapeptide angiotensin I (Ang I) [2,3]. Ang I is converted to angiotensin II (Ang II) by angiotensin-converting enzymes (ACE), expressed by the endothelial cells of several organs, such as lung, heart, kidney, and brain [4,5]. Ang II is the most relevant molecule of the RAS pathway and performs its function by activating the following G-protein-coupled receptors: angiotensin II receptor type 1 (AT1R) and angiotensin II receptor type 2 (AT2R) [6] (Figure 1).

The effects exerted by these two membrane receptors are opposite, in particular, AT1R induces detrimental effects, such as inflammation, fibrosis, and altered redox balance in addition to vasoconstrictive properties, whereas AT2R is involved in protective and regenerating actions (anti-inflammatory, anti-fibrotic, neurodegenerative, metabolic) and in the release of vasodilatory molecules [7,8,9]. Therefore, the equilibrium point of the RAS is represented by Ang II, which can also be converted into heptapeptide Ang-(1-7) thanks to the action of angiotensin-converting enzyme 2 (ACE2). Ang-(1-7), which can also be generated by the cleavage of ANG I by endopeptidases, and binds Mas receptors counteracting most of the deleterious actions of the ACE/Ang II/AT1 axis, especially in pathological conditions [10,11].

Due to the regulatory effects of ACE and ACE2 on the levels of Ang II, these peptidases are the main players in the regulation of blood pressure in the cardiovascular system [12,13].

Endothelial ACE2 overexpression functions as a negative regulator of the RAS, thus reducing blood pressure [14]. In an animal model, ACE2 cardiomyocyte overexpression seems to decrease the detrimental effects of hypertension and Ang II infusion [15]; the ACE2 pathway has been shown to exert different effects on cardiomyocytes in the heart [12,16,17]. Ang-(1-7) infusion can ameliorate myocardial performance, cardiac remodeling, and survival in an animal model of heart failure, exerting beneficial effects [18]. Other data have correlated ACE2 overexpression with cardiac fibrosis and arrhythmia [19,20].

## 2. RAS and Acute Lung Injury

Several sources of evidence suggest that the RAS represents an important target for the treatment of lung pathologies [2,21]. Indeed, the ACE/Ang II/AT1R axis plays a relevant role in promoting acute lung injury, while the ACE2/Ang-(1-7)/Mas pathway can antagonize and reduce pathological processes, including pulmonary hypertension and fibrosis [6,22,23,24,25,26].

Some data have demonstrated a connection between RAS and acute respiratory distress syndrome (ARDS) [4,27,28,29,30].

In experimental settings of acute lung injury, ACE2 deficient animals develop histological and functional ARDS [6]. In particular, Ang II is involved in a number of processes that take place in the lung, including the genesis of pulmonary edema due to regulation of pulmonary vasoconstriction and vascular permeability in response to hypoxia, stimulation of the lung production of inflammatory cytokines, induction of alveolar epithelial cells apoptosis, and fibroproliferation [27].

In 2003, during the SARS-related coronavirus (SARS-CoV) infection outbreak, a possible relation emerged between RAS and viral infections. This virus was characterized by a high mortality rate due to clinical respiratory failure linked to ARDS [31]. Intriguingly, ACE2 was shown to be a receptor for the SARS-CoV [32,33]. The SARS virus can enter the host cells through an endocytosis process mediated by the binding of its spike protein trimers with a hydrophobic pocket of the extracellular catalytic domain of ACE2 [34]. After virus entry, ACE2 levels decrease, thus enhancing Ang II release that may favor ARDS development [6,33]. In animal models, SARS-CoV infection in ACE2 knock-out mice reduces the development of lung injury [33]. Only SARS-CoV and human coronavirus NL63 were shown to bind ACE2 to invade host cells [35]. Moreover, overexpressing ACE2 in cell lines promoted efficient replication of SARS-CoV, while, when ACE2 is neutralized by antibodies, viral replication is inhibited [33]. 

In clinical studies, serum Ang II levels have been shown to be significantly elevated in patients with acute lung injury [36], and high serum Ang II levels have been associated with the severity and mortality of the infection [37]. While AT2R has the opposite effect of AT1R, AT2R agonists can reduce lung damage [6,38,39]. Ang-(1-7) and Mas receptor agonists alleviate acute lung injury in mouse models [39,40,41].

Some researchers also believe that blocking AT1R may reduce the response of inflammatory mediators and reduce acute lung injury compared to blocking ACE [40]. In particular, in animal models of lung injury, the ACE inhibitor captopril significantly reduced lipopolysaccharide (LPS) and induced detrimental changes in the lung by decreasing secretion of tumor necrosis factor α and interleukin 6, along with a reduction in Ang II/Ang-1-7 ratio and by reversing the increased ratio of ACE to ACE2 [41]. Those data are in agreement with recent evidence suggesting beneficial effects of anti-IL-6 receptor human antibody Tocilizumab for the treatment of SARS-CoV-2-induced ARDS [42]. Moreover, a recent study has shown that Coronavirus disease (COVID-19) patients with hypertension treated with ACE inhibitors and AT1R blockers had different values of viral load and an attenuation of the inflammatory response, likely through the inhibition of IL-6 levels [43].

## 3. Can RAS Targeting Be an Important Option for COVID-19?

SARS-CoV-2, responsible for Coronavirus disease (COVID-19), shares the same ACE2 viral receptor as SARS-CoV [44]. ACE2 is highly expressed in human lung tissue, but also in the nasal cavity and oral mucosa [10,45].

Currently, the majority of patients with COVID-19 clinical infection exhibit mild symptoms, such as fever, myalgia or fatigue, nasal congestion, sore throat, and cough [46,47]. A minority of patients rapidly develop complications, such as severe pneumonia, septic shock, and even clotting dysfunction.

At present, there is no effective treatment for COVID-19. The activation of the signaling pathway ACE2/Ang-(1-7)/Mas or the inhibition of the pathway ACE/Ang II/AT1R may be interesting from a therapeutic standpoint.

ACE inhibitors inhibit the formation of angiotensin II and, consequently, the effects triggered by its interaction with the receptors AT1R (vasoconstriction, sodium and water retention, sympathetic activation, cell growth) and AT2R receptors [47].

Previous studies have shown that SARS-CoV significantly reduces ACE2 in the lungs of infected mice [31,33].

The binding of COVID-19 and ACE2 seems to result in the exhaustion of ACE2, thus inhibiting the ACE2/Ang-(1-7)/Mas receptor pathway and altering the balance of the RAS, and this would lead to the exacerbation of acute severe pneumonia [48]. Although these and other authors believe in a detrimental effect of ACE inhibitors in COVID-19 patients, this assumption is still controversial, with other authors concluding that ACE inhibitors and AT1R inhibitors may reduce the lung inflammatory response and mortality and may be used in patients with COVID-19 pneumonia [48].

Moreover, the scientific community has recently focused its attention on AT1R inhibitors and their possible benefit for patients infected by COVID-19 who experience pneumonia [49], but even in this regard, there are still some doubts about the AT1R antagonists’ effects. Indeed, AT1R antagonists may intuitively increase the ACE2 expression [50,51], yielding harmful consequences for COVID-19 patients since ACE2 has been identified as the functional receptor for SARS-Cov2.

As mentioned above, after virus entry, ACE2 levels decrease; the reduction in ACE2 levels, on the one hand, would cause lower production of Ang- (1-7) from Ang II. On the other hand, it would cause an increase in unconverted Ang II, which, finding AT1R blocked by the drug, would act on AT2R, triggering a series of protective events on the lung, such as a reduction in vascular permeability and edema, reduction in pulmonary fibrosis processes. Furthermore, despite its reduction, a small amount of ACE2 remains able to convert Ang II to Ang-(1-7), allowing the conservation of the pulmonary protective mechanisms mediated by Mas receptors [6,33]. For these reasons, AT1R blockers may paradoxically represent a protective mechanism against pulmonary lesions from the virus.

With regard to ACE inhibitors, their action on ACE inhibits the formation of Ang II, thus preventing both the negative effects mediated by AT1R and the positive ones deriving from the Ang II binding with AT2R and from its transformation into Ang-(1-7). Ang-(1-7), however, can be produced by the degradation of Ang I by endopeptidase and ACE2 (with the intermediate formation of Ace-(1-9), Figure 1). Although ACE inhibition blocks the degradation of Ang-(1-9) to Ang-(1-7), Ang-(1-7) can still be produced directly from Ang I. In addition, ACE inhibitors prevent the degradation of Ang-(1-7) to Ang-(1-5) [1], increasing its levels. Therefore, as described for AT1R inhibitors, in this case, it would also seem that the protective effects on the lung could be preserved.

ACE2 is regulated by a gene that is located on the X chromosome [52], thus suggesting that some differences may exist in the expression of ACE2 between men and women.

Moreover, there is evidence that ACE2 tissue levels are regulated by estrogen.

Gupte, Thatcher et al. [53] demonstrated that high-fat-fed female mice exhibited elevated adipose ACE2 activity, and consequently increased plasma Ang-(1–7) levels, without affecting systolic blood pressure. Moreover, ovariectomy of HF-fed female mice, by reducing adipose ACE2 activity, promoted obesity and hypertension. On the contrary, HF-fed males had elevated systolic and diastolic blood pressure that were abolished by losartan [53]. We also know that estrogen in atrial myocardium modifies the local RAS homeostasis by increasing the ACE2-mRNA levels [54].

The deprivation of estrogen in post-menopause causes a loss of cardiovascular protection in women due to the possible shift from the ACE2/RAS axis to the ACE/RAS pathways [55]. Shoemaker et al. showed that adipocyte-derived ACE2 contributes to sex differences in obesity-hypertension through the regulation of the balance between Ang-(1-7) and Ang II, a benefit that is lost with estrogen deprivation in post-menopause [56].

If we consider that the susceptibility to SARS-Cov-2 infection should increase with increasing ACE2 levels, women should be more prone to the infection than men. In contrast, the epidemiological data available to date show gender-based clinical differences in the manifestations of SARS-CoV-2, with women getting slightly less ill than men [57] and a greater number of male patients [53], in particular elderly subjects, being affected by serious COVID-19 infections if compared with female patients. This information is in contrast with studies that show that the level of ACE2 decreases with age [57], and, therefore, appears to be higher in young people who generally develop a less severe form of COVID-2019. Given this apparent inconsistency, we can speculate that there are probably other factors that contribute to the severity of the disease.

Information about the ethnicity of the patients affected by COVID-19 will also be important to understand if African and Afro-American patients, who usually have low plasma renin levels, will be really less affected than other populations; this concept seems to be reinforced by data on COVID-19 available from World Health Organization at the moment and from our hundreds of cases of COVID-19 patients in the Abruzzo region, Italy (unpublished observations), although such observation may be biased by several temporal and season disease development-related issues.

Other evidence suggests that SARS-CoV-2 can cause myocardial injury, with the elevation of high-sensitivity cardiac troponin I levels [58] and also the mechanism underlying myocardial injury can be linked to the relationship between SARS-CoV-2 and ACE2 [59].

It should also be considered that the mortality rate is higher in patients with a history of high blood pressure, diabetes mellitus, and coronary heart disease [60]. ACE inhibitors and AT1R inhibitors withdrawal would not only prevent the antihypertensive effect but also the cardioprotective properties of these drugs.

## 4. Conclusions

Although the relationship between the RAS pathway and COVID-19 is not yet completely clear, ongoing mechanistic and observational studies are needed to clarify better the pathophysiology, as well as the efficacy and safety of therapeutic strategies targeting the RAS pathway in the treatment and/or prevention of the COVID-19 pandemia.

Until now, no evidence exists in support of the use of drugs that act on RAS in patients affected by COVID-19. As a matter of fact, the segment of the population affected by the most severe forms of COVID-19 is usually affected by arterial hypertension and has a history of heart disease [61], but we still have no information about their previous therapies and other relevant clinical aspects.

Moreover, a recent study has highlighted how SARS-CoV-2 cell entry depends on ACE2 and TMPRSS2, a serine protease. The interesting finding is that TMPRSS2 is blocked by a clinically approved protease inhibitor, and this opens up new scenarios from a therapeutic perspective [44].

There are many other research ideas that will allow us to conduct further studies in the next months with the aim of better clarifying any therapeutic strategies to defeat SARS-CoV-2 definitely, and the RAS will probably play a role as protagonist in this important challenge for medical scientists.
